# Evaluation of Mesna Co-administration in the Management of Acute Pancreatitis: A Pilot Randomized Clinical Trial

**DOI:** 10.5812/ijpr-168523

**Published:** 2026-05-04

**Authors:** Amir Sadeghi, Mohammad-Yousef Fazel, Mohammad Abbasinazari, Laleh Mahmoudi, Seyed-Mehregan Sadatsafavi, Parvaneh Mohammadi

**Affiliations:** 1 Gastroenterology and Liver Diseases Research Center, Research Institute for Gastroenterology and Liver Diseases, Shahid Beheshti University of Medical Sciences, Tehran, Iran; 2 Department of Clinical Pharmacy, School of Pharmacy, Shahid Beheshti University of Medical Sciences, Tehran, Iran; 3 Department of Clinical Pharmacy, School of Pharmacy, Shiraz University of Medical Sciences, Shiraz, Iran; 4 Health Policy Research Center, Institute of Health, Shiraz University of Medical Sciences, Shiraz, Iran

**Keywords:** Mesna, Pancreatitis, HAPS, MDA

## Abstract

**Background:**

The use of antioxidants has been shown to mitigate the symptoms of acute pancreatitis (AP) and improve associated biomarkers.

**Objectives:**

In light of the antioxidant properties of mesna, this pilot study was undertaken to determine whether the addition of mesna to standard treatment for AP offers superior outcomes compared with standard treatment alone.

**Methods:**

In a randomized open-label pilot trial, patients diagnosed with AP were enrolled in the study. Intravenous mesna at a dose of 400 mg/day was administered for 7 days. Clinical indicators, including the Harmless Acute Pancreatitis Score (HAPS), amylase, lipase, C-reactive protein (CRP), erythrocyte sedimentation rate (ESR), and malondialdehyde (MDA), were measured at baseline and after the 7-day treatment period.

**Results:**

Although MDA levels significantly decreased in the group receiving mesna combined with standard therapy compared with the group receiving standard therapy alone after 7 days (P = 0.01), no significant differences were observed between the 2 groups in terms of HAPS, amylase, lipase, CRP, or ESR.

**Conclusions:**

These findings suggest that the addition of mesna at a daily dose of 400 mg did not influence the overall outcome of AP after 7 days. Further large-scale studies with varying doses and durations of mesna administration are recommended.

## 1. Background

Acute pancreatitis (AP) is a serious and potentially fatal condition characterized by inflammation of the pancreas. It arises from the intracellular activation and release of inappropriate proteolytic enzymes, leading to active inflammatory responses and subsequent pancreatic tissue damage (1). Obstruction of the pancreatic bile duct, pancreatic ischemia, and the activation of inflammatory cytokines and pancreatic proteases are identified as the primary etiological factors in AP. The condition is characterized by a significant inflammatory response resulting from an imbalance between anti-inflammatory mechanisms and proinflammatory mediators (2). Reactive oxygen species (ROS) serve as critical signaling molecules that significantly contribute to the progression of inflammatory disorders. The increased generation of ROS by polymorphonuclear neutrophils at the site of inflammation leads to endothelial dysfunction and subsequent tissue injury (3).

In a meta-analysis conducted by Jeurnink et al, randomized controlled trials were assessed, focusing on the administration of antioxidants in the treatment of AP. The antioxidants evaluated in the study included glutamine, vitamin C, vitamin E, S-adenosylmethionine, and N-acetyl cysteine. Although the analysis indicated a potential benefit of glutamine in patients with AP, the authors emphasized the need for large-scale randomized trials to substantiate the effectiveness of antioxidants in managing AP (4). 2-Mercaptoethane sulfonate (mesna) is a synthetic compound with a sulfhydryl group, enabling it to effectively scavenge ROS. Mesna is extensively used to prevent hemorrhagic cystitis induced by cyclophosphamide and ifosfamide treatment (5).

Mesna has demonstrated antioxidant properties in a limited number of studies. Hagar et al conducted an animal study to investigate the potential effects of mesna in an experimental model of cerulein-induced AP. Their findings indicated that mesna significantly mitigated AP by reducing oxidative stress-related pancreatic damage and effectively preventing inflammation (6). Sadeghi et al reported that, in high-risk patients undergoing endoscopic retrograde cholangiopancreatography (ERCP), the combined administration of mesna and indomethacin had the potential to reduce both the incidence and severity of post-ERCP pancreatitis (7). Considering the limited evidence regarding the efficacy of mesna in mitigating AP, this pilot clinical trial aimed to assess whether mesna administration is more effective than standard treatment in reducing the severity of AP. The primary outcome was the Harmless Acute Pancreatitis Score (HAPS). Secondary outcomes included biochemical markers, namely serum amylase, lipase, C-reactive protein (CRP), erythrocyte sedimentation rate (ESR), and malondialdehyde (MDA).

## 2. Objectives

This pilot study aimed to determine whether the addition of mesna to standard treatment for AP offers superior outcomes compared with standard treatment alone.

## 3. Methods

### 3.1. Design

A randomized, open-label clinical trial was conducted. The study proposal was reviewed and approved by the Ethics Committee of the Midwifery, Nursing, and Pharmacy schools affiliated with Shahid Beheshti University of Medical Sciences (Approval No. IR.SBMU.PHARMACY.REC.1403.158). The trial was registered in the Iranian Clinical Trial Registry under Registration No. IRCT20121021011192N19. The trial was conducted in the gastroenterology and liver diseases ward of Taleghani Hospital, Tehran, Iran.

### 3.2. Patients and Outcomes

The inclusion criteria encompassed patients older than 20 years who had been diagnosed with AP, were hospitalized for treatment, and provided written informed consent to participate in the study. The exclusion criteria included any severe diseases affecting the primary visceral organs, including cardiac, hepatic, and nephrological conditions, as well as diabetes mellitus, autoimmune disorders, and tumors. The standard treatment protocol for AP was administered to all patients and comprised maintenance of water-electrolyte balance, provision of appropriate nutrition through total parenteral nutrition, pain management, and measures for infection prevention and treatment. Eligible patients were randomly assigned to 2 groups. Randomization was performed using a computer-generated block randomization method specific to each study center. In the experimental group, mesna (400 mg/day) was administered intravenously for 7 days, whereas the control group received standard treatment alone.

To assess the severity of AP, HAPS was used. HAPS is a diagnostic tool designed to predict with high accuracy which patients are likely to experience a mild course of pancreatitis. The scoring system is based on 3 parameters: the presence of signs of peritonitis, serum creatinine concentration, and hematocrit levels. A negative HAPS score indicates the absence of peritonitis, a serum creatinine concentration less than 2 mg/dL, and hematocrit levels below 43% for males or below 39.6% for females. Conversely, if any of these parameters is positive, the patient is categorized as HAPS-positive (8). HAPS scores (-/+) were determined at baseline and after 1 week in the participants. Pain severity was assessed before and after the study period (7 days). Blood samples were collected from all participants at baseline and again after a 7-day interval. The biochemical parameters analyzed included amylase, lipase, CRP, ESR, and MDA. All participants tolerated mesna well, and no individuals withdrew from the trial.

### 3.3. Statistical Analysis

All data were expressed as mean ± SD, when appropriate. Statistical Package for the Social Sciences version 22 (SPSS 22.0) was used for statistical analysis. The t-test, Mann-Whitney test, and chi-square test were used for the analysis, and P < 0.05 was considered statistically significant. Because this study was designed as a pilot trial, the sample size was determined based on a conventional rule-of-thumb approach, which recommends the inclusion of at least 30 participants. Considering the incidence rate of AP during the 4-month recruitment period at the study site, a target sample size of 40 - 45 patients was deemed feasible and appropriate.

## 4. Results

Over a duration of 4 months, a total of 43 patients were evaluated and successfully completed the trial (20 in the mesna group and 23 in the control group). The demographic characteristics of the participants are presented in Table 1. No significant differences were observed between the 2 groups in terms of mean age, sex distribution, body mass index, habitual history, or etiology of AP.

**Table 1.** Demographic Data of the Patients ^a^

Variables	Total Patients	Mesna Group (n = 20)	Control Group (n = 23)	P-Value
**Mean Age (y)**	56.3 ± 15.9	54 ± 15.8	58.3 ± 16.0	0.37
**Sex Distribution**				0.34
Female	17	6	11	
Male	26	14	12	
**Body Mass Index (kg/m²)**	25.6 ± 3.7	25.8 ± 3.6	25.5 ± 3.9	0.82
**Habitual History**				0.59
None	32	14	18	
Smoking	1	0	1	
Alcohol	3	1	2	
Opium	3	2	1	
Opium Plus Smoking	4	3	1	
**Cause of Acute Pancreatitis**				0.47
Biliary Stone	23	9	14	
Alcohol	2	2	0	
Post-ERCP	6	4	2	
Combination	8	3	5	
Unknown	4	2	2	

Abbreviation: ERCP, endoscopic retrograde cholangiopancreatography.

^a^ Values are presented as mean ± SD or No.

The HAPS scores of the participants were assessed and are presented in Table 2. Statistical analyses indicated no significant differences between the 2 groups in HAPS scores before and after the trial (P = 0.61 and P = 0.58, respectively).

**Table 2.** Harmless Acute Pancreatitis Score of the Participants Before and After the Trial in the Two Groups ^a^

Variables	Mesna Group Before Trial	Control Group Before Trial	P-Value	Mesna Group at End of Trial	Control Group at End of Trial	P-Value
**HAPS Score**			0.61			0.58
Negative	13 (30.2)	15 (34.9)		17 (39.6)	19 (44.1)	
Positive	7 (16.3)	8 (18.6)		3 (7)	4 (9.3)	

Abbreviation: HAPS, Harmless Acute Pancreatitis Score.

^a^ Values are presented as N (%).

Table 3 presents the levels of amylase, lipase, ESR, and CRP among the patients, as assessed both before and after the trial in the 2 groups. Statistical analysis demonstrated no significant differences in the mean levels of amylase, lipase, ESR, and CRP before the initiation of the trial (P = 0.09, 0.66, 0.64, and 0.86, respectively). Similarly, after 7 days, the mean levels of these parameters remained statistically comparable between the 2 groups (P = 0.35, 0.08, 0.74, and 0.91, respectively).

**Table 3.** Biochemical Parameters Before and After the Trial in the Two Groups ^a^

Variables	Mesna Group Before Trial	Control Group Before Trial	P-Value	Mesna Group at End of Trial	Control Group at End of Trial	P-Value
**Amylase (U/L)**	599 ± 664	655 ± 665	0.09	119 ± 112	135 ± 118	0.35
**Lipase (U/L)**	594 ± 671	633 ± 663	0.66	115 ± 178	273 ± 202	0.08
**ESR (mm/h)**	32 ± 24	25 ± 18	0.64	47 ± 30	41 ± 25	0.74
**CRP (mg/L)**	41 ± 50	46 ± 49	0.86	59 ± 55	56 ± 51	0.91

Abbreviations:CRP, C-reactive protein; ESR, erythrocyte sedimentation rate.

^a^ Values are presented as mean ± SD.

**Figure 1.**
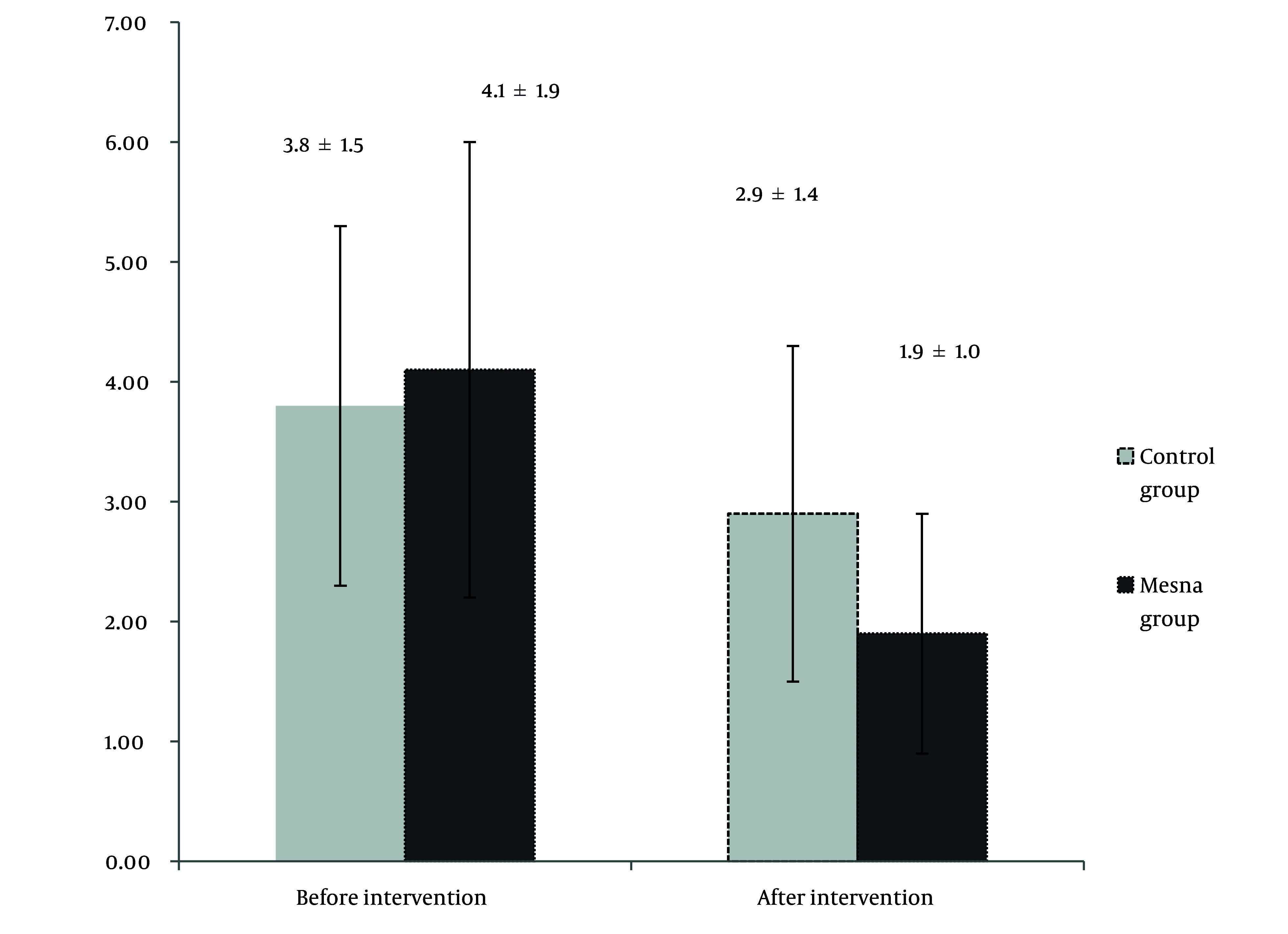
MDA levels before and after the trial in the two groups.

Figure 1 illustrates the levels of MDA in the 2 groups before and after the trial. At baseline, no statistically significant difference in MDA levels was observed between the 2 groups (P = 0.51). However, after 7 days, the MDA level in the mesna group was significantly lower than that in the control group (P = 0.01).

## 5. Discussion

Oxidative stress has been identified as a critical factor in the early stages of AP, with ROS altering signal transduction pathways regulated by redox mechanisms and causing direct oxidative damage. Previous research has highlighted the antioxidative effects of certain interventions (9, 10, 11). Building upon this foundation, the present study evaluated the impact of adding mesna to the AP treatment regimen in mitigating the severity of the disorder and its associated biomarkers. Markers of oxidative damage, including MDA and myeloperoxidase, increase, whereas levels of antioxidant markers, such as superoxide dismutase and glutathione peroxidase, decrease (9). Furthermore, a correlation has been observed between MDA concentration and the incidence of complications associated with AP (12). Accordingly, the present study measured MDA levels in the evaluated patients both at baseline and at the conclusion of the trial. Baseline MDA levels showed no statistically significant difference between the mesna group and the control group (P = 0.51). However, after 7 days, MDA levels were significantly reduced in the mesna group compared with the control group (P = 0.01), suggesting that mesna may effectively mitigate oxidative damage. It is important to note that other oxidative and antioxidative markers were not analyzed in this study. Future research involving a comprehensive assessment of these biomarkers may provide further insights into the potential effects of mesna on oxidative stress.

The HAPS is a straightforward and practical algorithm designed to predict the severity of AP in hospitalized patients. A HAPS score of 0 indicates that the patient does not require early aggressive interventions or advanced radiological evaluations during the initial stages of the disease (13). In our study, the HAPS scores of both groups improved after 7 days, with an increase of 9.4% in the mesna group and 9.9% in the control group, although this difference was not statistically significant. These findings suggest that standard management of AP, including pain relief, nutritional support, and fluid resuscitation, contributes to improved HAPS scores in both groups, whereas the addition of mesna does not appear to further enhance HAPS outcomes (P = 0.58).

Amylase and lipase, which are enzymes originating from pancreatic tissues, serve as fundamental biomarkers in the laboratory diagnosis of AP. The sensitivity and specificity of lipase surpass those of amylase in confirming AP. Notably, the concentration of lipase remains above the upper normal limit for approximately 7 - 14 days (14). In this study, amylase and lipase levels were measured on the seventh day, coinciding with the discontinuation of mesna. Following the management of AP in all participants, reductions in the levels of both amylase and lipase were observed after 7 days. However, no statistically significant differences were identified between the 2 groups in terms of the mean amylase and lipase levels on the seventh day (P = 0.35 and P = 0.08, respectively).

Because of its accessibility and affordability, CRP is the most commonly used single biomarker for assessing the severity of AP in contemporary clinical practice. A CRP concentration exceeding 150 mg/L is widely recognized as an indicator of severe AP (15). Notably, research conducted by Khanna et al demonstrated that a CRP level above 150 mg/L exhibits a sensitivity of 100% and a specificity of 81.4% for identifying pancreatic necrosis (16). In our preliminary investigation, the mean CRP levels remained below 100 mg/L across both groups and at both time points (before and after the intervention). This observation suggests that the AP cases examined in this trial were neither severe nor necrotizing, demonstrating consistency in AP severity among the participants. Furthermore, no significant difference in CRP levels was observed between the mesna and control groups by the seventh day of the study (P = 0.91). Given that the treatment protocol for AP was identical across both groups, it can be inferred that mesna did not influence the severity of the cases during this timeframe. ESR has demonstrated the capability to predict severe AP, albeit with slightly lower accuracy compared with CRP (17). In the present study, ESR levels remained consistent across the groups after 7 days, indicating that mesna administration did not exert a significant impact on ESR levels.

Although the combination of mesna (400 mg) and indomethacin has been shown to reduce the incidence of post-ERCP pancreatitis in high-risk groups (7), the findings of our trial suggest that the addition of mesna (400 mg/day) to routine management of AP significantly lowers MDA levels compared with routine management alone. However, no statistically significant differences were observed between the 2 groups in terms of the HAPS score or serum levels of amylase, lipase, CRP, and ESR. Furthermore, mesna administration was well tolerated, with no adverse reactions reported among the participants. This study serves as a preliminary investigation, and future research should consider administering higher doses of mesna over an extended duration to evaluate its potential therapeutic effects in mitigating AP. Additionally, because of constraints related to placebo preparation, the trial was conducted using an open-label design, which may have introduced potential bias. Future studies are recommended to adopt double- or triple-blind randomized controlled designs to minimize bias and enhance methodological rigor.

## Data Availability

The dataset presented in the study is available on request from the corresponding author during submission or after publication.

## Clinical Trial Registration Code

IRCT20121021011192N19.
